# Localization and Tumor Growth Inhibition of I-131-Labeled Monoclonal Antibody ERIC1 in a Subcutaneous Xenograft Model of Small Cell Lung Cancer in SCID Mice

**DOI:** 10.3390/ijms251910638

**Published:** 2024-10-02

**Authors:** Thomas Fischer, Christopher Dietrich, Felix Dietlein, Sergio Muñoz Vázquez, Beate Zimmermanns, Philipp Krapf, Ferdinand Sudbrock, Alexander Drzezga, Markus Dietlein, Klaus Schomäcker

**Affiliations:** 1 Department of Nuclear Medicine, Faculty of Medicine and University Hospital Cologne, University of Cologne, Kerpener Str. 62, 50937 Cologne, Germany; thomas.fischer@uk-koeln.de (T.F.); sergio.munoz-vazquez@uk-koeln.de (S.M.V.); beate.zimmermanns@uk-koeln.de (B.Z.); p.krapf@fz-juelich.de (P.K.); ferdinand.sudbrock@uk-koeln.de (F.S.); alexander.drzezga@uk-koeln.de (A.D.); markus.dietlein@uk-koeln.de (M.D.); 2 Institute and Polyclinic for Occupational Medicine, Environmental Medicine, and Preventive Research Faculty of Medicine and University Hospital Cologne, University of Cologne, Kerpener Str. 62, 50937 Cologne, Germany; christopher.dietrich@uk-koeln.de; 3 Computational Health Informatics Program, Boston Children’s Hospital, Harvard Medical School, Boston, MA 02115, USA; felix.dietlein@childrens.harvard.edu; 4 Forschungszentrum Jülich GmbH, Institute of Neuroscience and Medicine (INM-5), Wilhelm-Johnen-Straße, 52428 Jülich, Germany; 5 German Center for Neurodegenerative Diseases (DZNE), Bonn-Cologne, Venusberg-Campus 1/99, 53127 Bonn, Germany

**Keywords:** nuclear medicine, theranostics, SCLC, biokinetics, anti-NCAM antibody, ERIC1, I-131, mice, tumor growth inhibition

## Abstract

This study evaluates the efficacy of [^131^I]I-ERIC1 in targeting and inhibiting the growth of SCLC tumors in mice, focusing on tumor accumulation and regression and potential side effects. NCAM-positive NCI-H69 SCLC cells were implanted in CB 17 SCID mice, and [^131^I]I-ERIC1 biokinetics were measured in organs and tissues at four post-injection time points (24, 72, 96, and 120 h). The experimental series compared tumor growth, survival, and changes in blood counts among three treatment groups (1, 2, or 3 MBq) and a control group, with treatments initiated either two or five days post implantation. [^131^I]I-ERIC1 was synthesized with >95% radiochemical purity and a specific activity of 15 TBq/mmol. Tumor activity peaked at 31.5 ± 6.6% ID/g after four days, demonstrating significant antitumor efficacy, which resulted in sustained remission and extended survival. Hematological toxicity was observed, with the optimal dose identified as 2 MBq per animal administered two days post implantation. [^131^I]I-ERIC1 shows promise as a theranostic agent for personalized cancer treatment by effectively targeting SCLC tumors with manageable side effects. However, further studies are required to optimize dosing strategies and minimize toxicity.

## 1. Introduction

Lung cancer is the second most commonly diagnosed cancer worldwide, accounting for approximately 12% of annual global cancer cases. Small cell lung cancer (SCLC) represents nearly 13% of all lung cancers and has a disheartening five-year survival rate of around 7%. Patients diagnosed at an early stage have a more favorable five-year survival rate of nearly 30%. Approximately 70% of patients present with metastatic lesions at the time of diagnosis. The current standard treatment for SCLC includes chemotherapy, chemoradiotherapy, or chemotherapy combined with immunotherapy. However, most patients experience relapse and require second-line chemotherapy, which is associated with a very poor prognosis. This highlights the urgent need for new diagnostic and therapeutic tools to treat SCLC [1,2].

A new strategy that appropriately addresses this challenging issue and has been successfully applied in nuclear medicine is the use of theranostics. The term “theranostics” refers to the development of more specific, individualized therapies that combine diagnostic and therapeutic capabilities into a single pharmaceutical agent. Radiotheranostics is a tailored application of theranostics in nuclear medicine, using radionuclide-labeled substances (radiotheranostic agents). The success of this strategy requires both an appropriate carrier molecule and a target structure on or within the tumor cell. The therapeutic effect relies on a cytotoxic radioisotope (α particles, β particles, or Auger electron emitters), while the diagnostic component uses gamma-ray emissions, which can be detected by single-photon emission computed tomography (SPECT) or PET [3,4].

When considering potential targets, neural cell adhesion molecule 1 (NCAM1 (CD56)) emerges as promising. In 1976, Rutishauser et al. first described this surface molecule as number 56 in the “Cluster of Differentiation” (CD) [5]. NCAM1 (CD56) was subsequently detected in various tumors of neuroectodermal origin, such as neuroblastoma; rhabdomyosarcoma; various brain tumors; and, notably, small cell lung carcinoma [6,7,8].

Several authors [9,10,11,12] have reported that NCAM1 is expressed in the vast majority of small cell lung carcinomas.

Antibodies have proven to be effective vehicles for the targeting of NCAM1, and various antibody–drug conjugates have been developed to deliver cytotoxic substances specifically to small cell lung carcinoma cells via NCAM1 targeting [11,13,14].

In this study, a similar approach is employed, whereby radionuclide I-131 serves as the cytotoxic agent, forming the basis for an experimental theranostic approach [15].

Iodine-131 can be considered a theranostic agent because it possesses both therapeutic and diagnostic properties. As a radionuclide, I-131 emits beta particles, which exert a cytotoxic effect by damaging the DNA of targeted cancer cells, leading to cell death. Concurrently, I-131 emits gamma rays, which can be detected by imaging techniques such as single-photon emission computed tomography (SPECT). The gamma radiation emitted by I-131 allows for the measurement of the radionuclide in organ or tissue samples during animal experiments, without significant attenuation caused by interaction with matter. This dual functionality enables simultaneous tumor treatment and monitoring of the therapeutic response, making I-131 a prime example of a theranostic agent [16]. Decades of experience support this approach, particularly in the radioiodine therapy of benign and malignant thyroid diseases [17].

In our research group, antibody ERIC1 has proven to be a promising vehicle for the targeting of the NCAM1 receptor in tumor tissues. ERIC1 is an IgG1 immunoglobulin with κ light chains classified under Cluster I of monoclonal anti-NCAM antibodies. It exhibits high specificity for NCAM1 [18,19,20]. In our previous work using a mouse model with neuroblastomas, we demonstrated reversible, high-affinity binding of I-131-labeled ERIC1 to NCAM1, with K_d_ values of 9 × 10^−8^ M [21]. Notably, NCAM-positive tumors, particularly neuroblastomas, exhibited significant tumor accumulation and tumor growth inhibition after the application of this radioimmunoconjugate (RIC) [21,22]. This retention of radioactivity could be displaced by the co-administration of a cold antibody.

The present study aimed to investigate whether RIC [^131^I]I-ERIC1 demonstrates similarly impressive performance in SCLC, particularly in terms of tumor accumulation and antibody-mediated radiation effects on tumor growth.

## 2. Results

### 2.1. Radioactive Antibodies

Labelling of ERIC1 with I-131 achieved a yield of approximately 50–60%, with radiochemical purity exceeding 95%. For [^131^I]I-ERIC1, the deiodination rate was less than 5% over 6 h in saline solution. A specific activity of approximately 15 TBq/mmol was obtained. Quality control, which was performed using size-exclusion high-performance liquid chromatography (HPLC), showed that the labeled antibody eluted after 6 to 7 min, while free [^131^I]I-iodide peaked at 10 to 11 min, confirming a radiochemical purity greater than 95%.

### 2.2. Biokinetics

The decay-corrected activities of individual organs and tissues were expressed as a percentage of the total administered activity per gram of organ weight (% ID/g) for comparison. The biodistribution of RIC is presented as the percentage of the administered dose per gram of organ or tissue relative to the administered activity and expressed as arithmetic means with standard deviations (% ID/g ± SD) (Table 1).

One day after RIC administration, the blood contained the highest percentage of the administered activity (31.7 ± 4.7% ID/g)—nearly double that of the tumor (17.5 ± 2.6% ID/g). However, at this time point, the xenograft had already accumulated more activity than other organs, including the heart, lungs, and thyroid, all of which exhibited values exceeding 10% ID/g.

After three days, the activity in the tumor (21.2 ± 3.9% ID/g) was nearly equal to that in the blood (22.5 ± 0.8% ID/g), which had decreased by about one-third from the 24 h value. Other organs also showed reduced accumulation, with thyroid activity nearly halving from 10.8 ± 6.7% ID/g to 5.7 ± 0.6% ID/g.

Another 24 h later, the activity in the tumor peaked at 31.5 ± 6.6% ID/g. Slight changes in activity were observed in other organs, with some showing increases and others showing decreases. Notably, urine activity significantly increased from 2.7 ± 0.9% ID/g at 24 h and 1.9 ± 1.6% ID/g at 72 h to 5.4 ± 1.5% ID/g.

At the final measurement time point, 120 h post injection, tumor accumulation had slightly decreased to 28.9 ± 6.0% ID/g. In other organs, activities largely decreased compared to the 96 h values. However, increases in activity were observed in both the blood and femur, with femur activity approaching the baseline value (3.8 ± 0.6% ID/g compared to 4.1 ± 0.6% ID/g).

### 2.3. Therapeutic Effects

#### 2.3.1. Tumor Size

To achieve variation in initial tumor volumes, treatments with the RIC were administered on days 2 and 5 after tumor implantation. This allowed for an assessment of the treatment’s efficacy at different stages of tumor growth.

**Figure 1:**
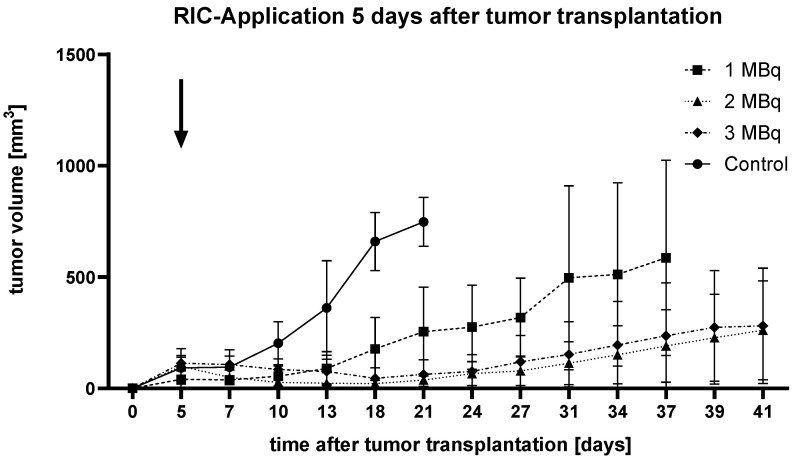
Graph depicting the change in tumor volume over time after tumor transplantation in female SCID mice (N = 5) bearing SCLC treated with [^131^I]I-ERIC1 at three different doses (1, 2, and 3 MBq) compared to an untreated control group. RIC administration occurred 5 days after tumor transplantation (indicated by an arrow). Error bars represent the standard error of the mean.

- RIC treatment was administered **five days** after tumor transplantation (Figure 1).

Only 5 days after tumor implantation (indicated by an arrow in Figure 1), the control group began to show tendencies of accelerated tumor growth compared to the therapy groups. However, a statistically significant difference could only be established for the 2 MBq group using the Kruskal–Wallis test with Dunn’s multiple comparison test (*p* = 0.008). After 18 days, the 2 MBq (*p* = 0.0028) and 3 MBq (*p* = 0.0329) groups showed a statistically significant reduction in tumor growth compared to the control group. This remained the case up to 21 days after tumor implantation (2 MBq: *p* = 0.0033, 3 MBq: *p* = 0.0324). At no point during the study were there significant differences observed within the treatment groups.

Since statistical significance between individual treatment groups and the control group was only observed for specific conditions and no significant differences were detected within the treatment groups, we decided to reorganize the data. At each time point after RIC administration (2 days post tumor implantation), only two groups were considered, namely the control group and a combined treatment group that included results from the 1 MBq, 2 MBq, and 3 MBq groups. To determine if there was a significant difference at any specific time point after RIC administration, we used the Mann–Whitney U test (Wilcoxon rank-sum test).

This approach confirmed the results of the Kruskal–Wallis test. Statistically significant differences between the treatment group (N = 15) and the control group (N = 5) were observed as early as 5 days after RIC administration (*p* = 0.0025). These differences persisted until the termination criterion was reached in the control animals.

Ultimately, tumor size in the control group reached the endpoint in all animals within three weeks, with tumors being more than 20 times larger than those in the 2 MBq group.

**Figure 2:**
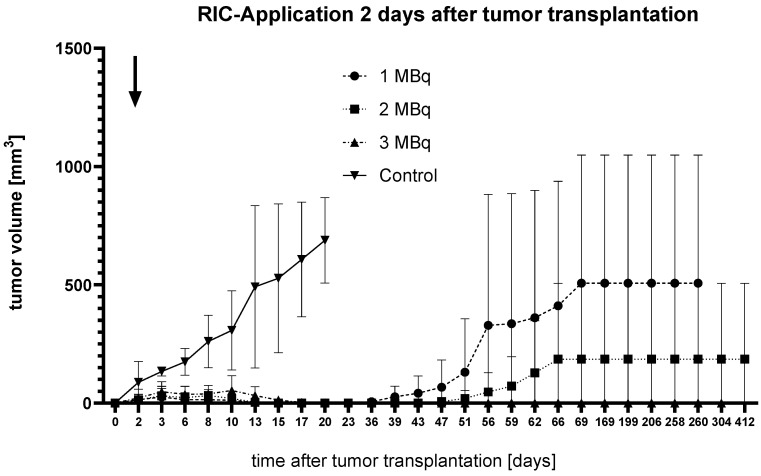
Graph showing the change in tumor volume over time after tumor transplantation in female SCID mice (N = 4) bearing SCLC treated with [^131^I]I-ERIC1 at three different dose levels (1, 2, and 3 MBq) compared to an untreated control group. RIC administration occurred 2 days after tumor transplantation (indicated by an arrow). Error bars represent the standard error of the mean.

- RIC Administration **Two Days** After Tumor Transplantation (Figure 2).

In the second series, tumor volumes were similar across all groups until the day of [^131^I]I-ERIC1 administration. The control group experienced rapid tumor growth, leading to complete progression in all animals within three weeks. In the treatment groups, tumor growth stagnated for one week post treatment, then went into complete remission.

In the 1 MBq group, tumor regrowth occurred after approximately six weeks. In the 2 MBq group, regrowth occurred in one mouse after seven weeks, while the other animals remained in remission. In the 3 MBq group, no tumor regrowth was observed throughout the 412-day observation period.

Clear signs of accelerated tumor growth in the control group, compared to all therapy groups, became evident six days after RIC administration in the group where RIC was applied two days after tumor transplantation. However, statistical significance was only confirmed for the 3 MBq group (*p* = 0.0468) using the Kruskal–Wallis test. Similarly, on day 11 post administration, statistical significance was only demonstrated in the 1 MBq group (*p* = 0.02).

To further analyze the results, the data were reorganized to compare the combined treatment group (N = 14) and the control group (N = 12) at each time point using the Mann–Whitney U test (Wilcoxon rank-sum test). Under this approach, a statistically significant difference between the treatment and control groups emerged as early as two days after RIC administration (*p* = 0.0005). This difference persisted until the control group reached the endpoint criterion at approximately three weeks.

In the 1 MBq group, after tumor regression (no visible tumor) from day 15 to day 23, tumor regrowth was observed, with the endpoint criterion reached around 260 days after tumor implantation. In the 2 MBq group, tumor regression occurred on day 17 and persisted until day 43, with regrowth leading to the endpoint around 412 days after implantation. In the 3 MBq group, tumor regression was observed after 23 days, and no regrowth was observed over the 412-day observation period. After tumor regrowth, there was no significant difference in tumor volumes between the 1 MBq and 2 MBq groups.

#### 2.3.2. Survival

**Figure 3:**
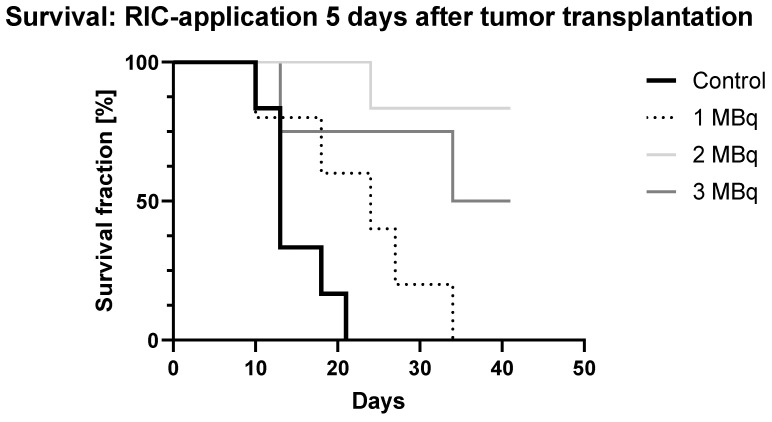
Survival analysis (Kaplan–Meier, GraphPad Prism 10.2.2) over time following RIC administration in female SCID mice (N = 5) bearing SCLC treated with [^131^I]I-ERIC1 at three different doses (1, 2, and 3 MBq) compared to an untreated control group. RIC administration occurred 5 days after tumor transplantation.

- Administration of the RIC **Five Days** After Tumor Transplantation (Figure 3).

Survival analysis (Kaplan–Meier, GraphPad Prism 10.3.0) was performed over time following RIC administration in female SCID mice (N = 5) bearing SCLC treated with [^131^I]I-ERIC1 at three different doses (1, 2, and 3 MBq) compared to an untreated control group, with RIC administration occurring 5 days after tumor transplantation.

In the Kaplan–Meier survival analysis, 80% of the control group had died by day 18. Three days later, the last control mouse reached the tumor-size termination criterion, resulting in significantly lower survival compared to the treatment groups (*p* ≤ 0.0152), as only 21.7% of the treated mice had died by that time.

Within the treatment groups, the 1 MBq group showed a continuous decline from day 13, with complete mortality by day 37. Deaths occurred on days 13 and 27, with the remaining mice reaching the tumor size criteria. Compared to the minimally affected 2 MBq group, the 1 MBq group had significantly lower survival (*p* = 0.0042), although it narrowly missed statistical significance compared to the 3 MBq group (*p* = 0.808).

By week three, 75% of the 3 MBq group was still alive, although one tumor-free mouse died on day 18. Another mouse died due to reaching the tumor size criterion by the study’s end, leaving 50% surviving for nearly six weeks. The 2 MBq group had the fewest losses, with only one tumor-free mouse found dead on day 27. By the end of the study, 83% of the 2 MBq group remained alive.

**Figure 4:**
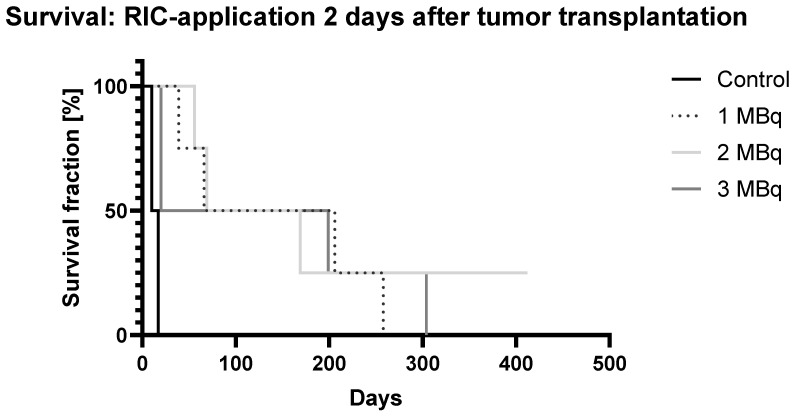
Kaplan–Meier survival analysis (GraphPad Prism 10.2.2) over time following RIC administration in female SCID mice (N = 5) bearing SCLC treated with [^131^I]I-ERIC1 at three different dose levels (1, 2, and 3 MBq) compared to an untreated control group. RIC was administered 2 days after tumor transplantation.

- Administration of the RIC **Two Days** After Tumor Transplantation (Figure 4)

The control group’s survival ended approximately three weeks post implantation, similar to the results of the first experiment. Half of the control mice reached the tumor-size endpoint by day 13, with the remaining half reaching the endpoint by day 20.

In the treatment groups, administering the RIC two days after xenograft implantation resulted in a higher six-week survival rate of 83.3%, compared to 53.3% with later RIC administration. Only the 3 MBq group lost members during this period, with two mice found dead on day 23 without tumors. In the 2 MBq group, one mouse died on day 65 due to tumor size—a significant delay compared to the first experiment.

The most notable difference was observed in the 1 MBq group, which survived nearly eight weeks, whereas all mice had died by five weeks in the delayed RIC administration group, showing a three-week survival benefit from earlier RIC administration. At the end of the observation period, two of the four 1 MBq mice reached the critical tumor size, while the remaining 50% were tumor-free and alive at ten weeks.

Prolonged observation revealed the following two key points: long-term, tumor-free survival remained rare, despite treatment, as survival curves gradually declined over several months. However, this decline was less pronounced in treatment groups than in controls, particularly with earlier RIC administration. Following the 69-day observation period, which ended with the death of the last tumor-bearing mouse, a nearly year-long follow-up showed that 58.3% of the mice remained tumor-free. This rate held for nearly 100 days until two deaths occurred in the 2 MBq group on days 169 and 199. The last mouse in this group outlived the others by over 100 days, remaining healthy at the 412-day endpoint.

#### 2.3.3. Side Effects

- 1. Leukocyte count

At no time point after RIC administration did the described statistical tests demonstrate any significant difference between the control group and the treatment groups or within the treatment groups themselves. Only when the values were combined into a single treatment group and a control group—regardless of the time points post RIC administration and the administered radioactivity—was a significant difference in leukocyte count detected using the Mann–Whitney U test. The leukocyte count in treated animals was (3026 ± 261.5)/μL, compared to (4773 ± 741)/μL in control groups (*p* = 0.0293).

- 2. Erythrocyte Count

Statistical tests at various time points revealed no significant difference between the treatment groups and the control groups or within the treatment groups themselves. Even when analyzing all treatment groups together, regardless of administered radioactivity and time post RIC administration, no statistically significant difference was observed between the treated animals and the control group (erythrocyte count in treated animals: (6.600 ± 0.410) × 10^6^/μL; erythrocyte count in control groups: (7.510 ± 0.458) × 10^6^/μL; *p* = 0.3595).

- 3. Thrombocyte count

Analysis conducted using the above-described statistical methods showed no significant differences between the treatment groups and the control group or within the treatment groups at any time point after RIC administration. However, when all treatment groups were compared to the control group, regardless of the time after RIC administration and the administered radioactivity, a significant decrease in platelet count was observed in the treated animals (platelet count in treated animals: (0.565 ± 0.32) × 10^3^/μL platelet count in control groups: (0.771 ± 0.63) × 10^3^/μL; ***p*** = 0.0079).

## 3. Discussion

Our work on the therapeutic efficacy of [^131^I]I-ERIC1 builds upon key prior knowledge derived from our studies on the biokinetics and efficacy of the RIC in neuroblastoma [8,21,23,24]. This foundation allowed us to anticipate that [^131^I]I-ERIC1 would exhibit high tumor affinity for NCAM-positive SCLC, as had already been demonstrated in NCAM-positive neuroblastoma. Additionally, we knew that the RIC binds reversibly to NCAM, characterized by K_D_ values of approximately 10^−7^), and that the radioactive antibody can be displaced from tumor tissue by non-radioactive ERIC1. We also knew that cold antibody or [^131^I]I-iodide alone is ineffective and that the radiotoxicity of [^131^I]I-ERIC1, with the first signs of radiation toxicity in healthy SCID mice, occurs at around 5 MBq per animal. Weight reduction causally linked to the RIC was only observed at doses of 16–22 MBq per animal. Furthermore, the optimal dose for achieving significant tumor reduction, which also resulted in a five-fold increase in survival time compared to an untreated control group, was identified as a single dose of 2–3 MBq per animal.

Biokinetic studies in mice with small cell lung carcinoma conducted using [^131^I]I-ERIC1 demonstrated high radiochemical purity, tumor specificity, and significant accumulation within 24 h, peaking at 96 h. However, considerable non-tumor radioactivity in the blood and highly perfused organs posed risks of side effects and complicated its potential therapeutic application. Despite these challenges, the investigation into [^131^I]I-ERIC1’s potential for treating small cell lung carcinoma (SCLC) continued for several compelling reasons.

The high tumor specificity and accumulation indicate that the antibody effectively targets and delivers the radioactive payload to tumor cells, a crucial factor for successful cancer therapy. The decrease in tumor activity following the peak suggests a therapeutic effect, leading to tumor mass reduction and showing promise in reducing tumor size. Although high background activity is common in radioimmunotherapy (RIT) [25,26,27], it highlights the need for innovative strategies to manage non-specific accumulation. This ongoing challenge is an area of active research, with potential solutions including modifications to the antibody structure, co-administration with agents that block non-specific binding, or the development of novel delivery systems to enhance tumor specificity [28,29].

Experience with [^131^I]I-ERIC1 in neuroblastoma has also showed encouraging therapeutic effects with manageable side effects, despite considerable extratumoral accumulation. This justified exploring the therapeutic potential of the non-optimized antibody, with the anticipation that promising results could be further enhanced through optimization.

This study provides valuable insights into the evaluation of [^131^I]I-ERIC1’s efficacy, safety, and side effects, which serve as crucial criteria for the assessment of the viability of a radiopharmaceutical theranostic agent.

- Efficacy:

While [^131^I]I-ERIC1 exhibited promising therapeutic effects, its safety profile warrants careful consideration. The highest dose was linked to earlier mortality, indicating dose-dependent toxicity. Notably, leukocyte depletion occurred at this dose, with incomplete recovery even after four weeks, raising concerns about potential myelotoxicity. However, the lower dose demonstrated an optimal balance between efficacy and safety, achieving high survival rates with fewer side effects, suggesting that with appropriate dosing, [^131^I]I-ERIC1 can be safely administered.

- Safety:

While [^131^I]I-ERIC1 exhibited promising therapeutic effects, its safety profile requires careful consideration. The highest dose was associated with earlier mortality, indicating dose-dependent toxicity. Leukocyte depletion was notable at this dose, with incomplete recovery even after four weeks, highlighting potential myelotoxicity. However, the lower dose demonstrated an optimal balance between efficacy and safety, achieving high survival rates with fewer side effects, suggesting that, with appropriate dosing, [^131^I]I-ERIC1 can be administered safely.

- Side Effects:

The primary observed side effects were hematological—specifically, myelotoxicity. Challenges in blood sample collection procedures might have contributed to variability in the findings, but the trends indicate a clear impact on the hematopoietic system. These side effects emphasize the need for careful dose management and monitoring of blood parameters during treatment.

- Theranostic Potential:

[^131^I]I-ERIC1 shows promise as a theranostic agent due to its dual capabilities in diagnostics and therapy. Its high tumor specificity and strong accumulation make it suitable for both tumor detection and the delivery of targeted radiotherapy. The ability to achieve temporary or complete remission with early intervention and appropriate dosing supports its therapeutic efficacy. Moreover, its diagnostic potential can be leveraged to monitor tumor response and adjust treatment plans in real time, enhancing personalized cancer treatment strategies.

### Limitations of the Study

One limitation of the present study is the use of a murine antibody (ERIC1), which may limit direct translational applicability, particularly due to potential immunogenicity in humans. While the findings demonstrate the promising theranostic potential of the radioiodinated antibody in preclinical models, the efficacy, biodistribution, and safety of a humanized version of the antibody will need to be reassessed in future studies. Additionally, the immune response to murine antibodies may differ significantly from that of humanized antibodies, necessitating further investigation to validate these findings in a clinical context. We recognize this as a crucial step toward the advancement of the approach to clinical application.

Another limitation of our study is the exclusive use of the H146 cell line in both in vitro and in vivo experiments. While H146 is a widely studied SCLC cell line, its growth characteristics—such as growing in suspension, forming aggregates, and its sensitivity to handling—may introduce variability in experimental outcomes. Furthermore, the absence of NCAM-negative SCLC cells and normal lung epithelial controls limits the scope of our findings. Nonetheless, this study provides essential proof of concept for the use of [^131^I]I-ERIC1 in targeting NCAM-positive SCLC. Future studies will include a broader panel of SCLC cell lines, as well as appropriate controls, to ensure more robust conclusions regarding the efficacy of [^131^I]I-ERIC1.

We are also aware that the number of animals used (N = 4–5) is relatively small. Larger group sizes would likely provide more robust statistical power. However, we aimed to design the study in a way that balances the collection of meaningful data with the minimization of animal use, in accordance with ethical guidelines for animal research. Despite the small group sizes, we believe the results are valid, as the observed effects, particularly the differences between the treatment and control groups, are both plausible and statistically significant.

## 4. Materials and Methods

### 4.1. I-131

[^131^I]Iodine in the form of sodium iodide (7.4 GBq/mL; 370 MBq in a 50 μL NaOH solution) was obtained from Covidien GmbH, Neustadt, Germany.

### 4.2. Antibody

ERIC1 is a murine monoclonal antibody of the IgG1 isotype that recognizes the 140 and 180 kDa isoforms of NCAM, the most common isoforms in neuroblastomas. ERIC1 antibodies were purified from serum-free hybridoma supernatants using Protein-G affinity chromatography, following the method described by Jensen et al. [26].

### 4.3. Labelling with I-131

The ERIC1 antibody was radiolabeled with I-131 using a variation of the chloramine-T method [21], in which chloramine-T solution (Merck KGaA, Darmstadt, Germany) is used as an oxidant. To a 100 µL solution of the antibody (0.06 mg = 0.6 mg/mL), 50 µL phosphate buffer (0.25 M; pH 7.5, PAA Laboratories GmbH, Cölbe, Germany), 15 µL [^131^I]NaI (105 MBq), and 25 µL chloramine-T solution (1.4 mg/mL) were added. After 1 min of incubation at room temperature, 50 µL NaHSO_3_ (2.8 mg/mL, (Merck KGaA, Darmstadt, Germany)) was added to stop the reaction. After labeling, the radiolabeled antibody was purified to remove free I-131, remnants of the buffer, and residual chloramine-T solution.

An NAP-5 column (Amersham Biosciences; Piscataway, NJ, Sephadex G-25 medium) was used for purification. Quality control was performed using size-exclusion HPLC (Toso-Haas TSKgel 2000 SK column, Tosoh Bioscience GmbH, Griesheim, Germany; HPLC system: Knauer, Berlin, Germany; radioactivity detector: model ‘steffi’, Raytest, Straubenhardt, Germany). The in vitro stability of [^131^I]I-ERIC1 was assessed using HPLC six hours after the labeling procedure (for a representative HPLC radiochromatogram, see Supplementary Materials file).

### 4.4. Cell Line

NCI-H69 SCLC cells (CLS Cell Lines Service GmbH, Eppelheim, Germany) were incubated at 37 °C with 5% CO_2_ and suspended in 30 mL of medium. The cells formed small aggregates near the bottom of the culture flasks. The medium was changed every two days, and subculturing was required approximately once a week. The medium consisted of RPMI 1640, fetal calf serum (FCS), and antibiotics/antimycotics (PAA Laboratories GmbH, Cölbe, Germany). Cells were passaged when necessary to maintain optimal cell density. Regular macroscopic and light microscopy (Olympus GmbH, Hamburg, Germany) evaluations were conducted to check for contamination and assess cell viability. NCAM expression on NCI-H69 SCLC cells was detected by flow cytometry as previously described [21].

For cryopreservation, cells were frozen in a medium containing FCS and DMSO and stored at −80 °C. For reuse, cells were thawed, centrifuged, and resuspended in fresh medium, with the first medium change occurring 24 h later to remove DMSO (Sigma-Aldrich GmbH, Taufkirchen, Germany).

Cell counts were performed by diluting the cell suspension, staining with trypan blue, and counting cells on a hemocytometer (Merck KGaA, Darmstadt, Germany). The total number of cells per milliliter was calculated.

### 4.5. Animal Experiments

Female SCID mice (6 to 8 weeks old) lacking T and B lymphocytes but with active NK cell systems were used as experimental animals.

All animals were purchased from Charles River Laboratories International, Inc. (Wilmington, NC, USA). All animal experiments were conducted in compliance with the German Animal Protection Law under the supervision of the local Animal Welfare Officer. The use of radioactivity, including antibody labeling and its application in animal experiments, was conducted with the appropriate permissions. Specifically, approval was granted by the Regional Government of Cologne (approval number: 50.203.2-K 19, 15/05) under the project titled, “Investigation of the biodistribution and therapeutic efficacy of the radiolabeled anti-NCAM antibody ERIC1 in small cell lung cancer”.

Tumor transplantation was performed by shaving the right flank of each mouse with a long-hair trimmer. Approximately 24 h before implantation, the mice received NK cell antibody Anti-ASIALO-GM1 (20 μL = 0.02 mg Anti-ASIALO-GM1 (Wako Chemicals GmbH, Neuss, Deutschland) per 20 μL solution per mouse) to deplete NK cells. [30]. To facilitate intravenous injection, mice were exposed to red light to promote tail-vein dilation. Subcutaneous xenografts were then injected into the right flank (1 × 10⁷ cells in 100 μL of 0.9% NaCl, PAA Laboratories GmbH, Cölbe, Germany). H69 cell suspensions were prepared just before injection by centrifugation, resuspension, and loading into insulin syringes to ensure accurate dosing.

All animal experiments adhered to the following termination criteria: euthanasia by cervical dislocation performed by a veterinarian if the mice exhibited visible suffering or if the tumor diameter reached ≥10 mm. Deceased animals were immediately frozen after euthanasia or upon discovery.

### 4.6. Biokinetics

Biokinetic studies of RIC [^131^I]I-ERIC1 were conducted at the following four time points with five mice each: 24, 72, 96, and 120 h after intravenous injection. For the 24 and 72 h groups, approximately 0.25 MBq of the radiolabeled antibody in 30 μL (8.3 MBq/mL) was administered 13 days after xenograft implantation. For the 96 and 120 h groups, approximately 1 MBq in 40 μL (25 MBq/mL) was administered 11 days after implantation.

The rationale for using the higher activity was to ensure measurable radioactivity levels in small organ samples with sufficient statistical accuracy over the extended study duration of up to 120 h, taking into account the half-life of I-131 (8.02 h). This approach allowed for more reliable data collection over longer time points.

After the specified time points, the mice were euthanized, and various organs and tissues were collected for analysis. The measured activity was corrected and expressed as a percentage of the injected dose per gram of organ weight (% ID/g).

### 4.7. Influence of Radioactivity on Tumor Growth

To evaluate the therapeutic potential of [^131^I]I-ERIC1 in SCLC, the effects and side effects of different radioactive doses were tested. After subcutaneous injection of 1 × 10⁷ H69 cells, mice were divided into the following four groups: three treatment groups and one untreated control group. Additional control groups received either the non-radioactive antibody or [^131^I]I-iodide alone.

In the first series, five days post implantation, five mice in each treatment group received a single intravenous dose of 1, 2, or 3 MBq of the radiolabeled antibody in 50 μL. In the second series, after only two days of tumor growth, four mice per group received the same doses.

Tumor diameters were measured every two to three days using a micrometer screw gauge after shaving the right flank of the mice. Tumor volumes were calculated assuming a spherical shape to provide a statistical representation of growth.

During the experiment, both planned euthanasia and spontaneous deaths occurred, either with or without tumor burden. To avoid statistical bias, tumor size was recorded on the day of death and used for analysis until all mice in the group had died.

Tumor growth and the resulting growth delay—defined as the time to regain the tumor size at the start of therapy—were the primary criteria for evaluating treatment success. Survival outcomes were interpreted based on the efficacy and side effects of [^131^I]I-ERIC1 therapy.

### 4.8. Radiation-Induced Side-Effects of [^131^I]I-ERIC1

Parallel to the investigations of the impact of different [^131^I]I-ERIC1 radioactivities on tumor growth, radiation-induced side effects of the RIC were assessed in the same test animals by comparing their blood counts to those of the control group. For this purpose, the tail tip of each test animal was resected.

The blood count data presented here as a function of time after RIC application were obtained from blood samples of groups that received RIC both 5 days and 2 days after tumor transplantation. This approach aimed to minimize the distress of the experimental animals due to the complexity of the sample collection process. Consequently, it was not possible to determine whether the time interval between tumor implantation and RIC application had an impact on the blood count. In the first experimental series (RIC application 5 days after tumor implantation), duplicates were examined for each experimental condition.

The resulting blood drop was collected using a heparin-coated capillary (Sarstedt AG & Co., Nümbrecht, Germany). Approximately 30–40 µL of blood was collected in an EDTA test tube and analyzed using a COULTER^®^ Ac·T diff™ Analyzer (Beckman Coulter GmbH, Krefeld, Germany), determining the number of erythrocytes, leukocytes, and thrombocytes in the blood count. Each measurement was carried out in duplicate.

### 4.9. Experimental Design

Table 2 describes the administered activities, injection volumes, and radiochemical concentrations of the RIC [^131^I]I-ERIC1 in MBq/mL that were used for the respective experimental inquiries.

### 4.10. Statistics

To analyze the effects of varying doses of a therapeutic agent on tumor volume in tumor-bearing animals, we employed the Mann–Whitney U test (Wilcoxon rank-sum test) for comparisons between the treatment and control groups at specific time points. Given the small sample sizes (N = 4 or 5 per group) and the non-normal distribution of the data, this non-parametric test was chosen for its robustness in handling such data without assuming normality.

We initially considered using the Kruskal–Wallis test for comparison of multiple treatment groups, but after further analysis, we decided to focus on pairwise comparisons for a more direct assessment of treatment effects. As a result, we combined the results of the 1 MBq, 2 MBq, and 3 MBq groups into a single treatment group for each time point after RIC administration, allowing for a straightforward comparison with the control group.

GraphPad Prism 10.3.0 (GraphPad Software, Boston, MA 02110, USA) was used for all statistical analyses, which provided a user-friendly interface for performing the Mann–Whitney U test and ensuring accurate evaluation of our data.

## 5. Conclusions

[^131^I]I-ERIC1 exhibits strong efficacy in targeting and reducing SCLC tumors, particularly with early administration. While its safety profile is dose-dependent, it can be managed through careful monitoring and dose optimization. The primary side effects are hematological but generally transient. Given its dual diagnostic and therapeutic capabilities, [^131^I]I-ERIC1 holds significant potential as a theranostic agent, offering a promising approach for personalized cancer treatment. Further research is warranted to refine dosing strategies and enhance its therapeutic index, ensuring maximum benefit with minimal adverse effects.

## Figures and Tables

**Table 1:** Changes in radioactivity concentrations in organs and tissues (% ID/g ± SD) at various times after the administration of [^131^I]I-ERIC1 in female SCID mice (N = 5) bearing SCLC.

Organs	Activity Concentration [% ID/g ± SD]			
	24 h p.i.	72 h p.i.	96 h p.i.	120 h p.i.
Blood	31.7 ± 4.7	22.5 ± 0.8	22.3 ± 5.2	24.0 ± 2.8
Liver	6.1 ± 0.7	4.5 ± 0.2	4.1 ± 1.2	3.6 ± 0.7
Spleen	8.4 ± 1.3	6.2 ± 0.6	7.3 ± 1.6	5.4 ± 1.7
Kidneys	6.5 ± 0.4	4.9 ± 0.1	5.2 ± 1.4	5.1 ± 0.7
Muscle	2.7 ± 0.7	2.8 ± 0.4	1.7 ± 0.5	1.7 ± 0.3
Femur	4.1 ± 0.6	3.2 ± 0.3	2.7 ± 0.5	3.8 ± 0.6
Thyroid	10.8 ± 6.7	5.7 ± 0.6	7.1 ± 4.8	5.3 ± 1.1
Lung	12.0 ± 2.3	9.5 ± 1.3	9.9 ± 2.6	10.5 ± 1.4
GIT	3.3 ± 0.4	2.3 ± 0.2	2.0 ± 0.6	1.5 ± 0.8
Tumor	17.5 ± 2.6	21.2 ± 3.9	31.5 ± 6.6	28.9 ± 6.0
Urine	2.7 ± 0.9	1.9 ± 1.6	5.4 ± 1.5	5.4 ± 4.9
Heart	13.4 ± 10.8	9.9 ± 2.0	10.9 ± 9.5	8.9 ± 1.0

**Table 2:** Administered activities, injection volumes, and radiochemical concentrations of the RIC [^131^I]I-ERIC1 in MBq/mL used for the respective experimental inquiries.

Aim		Radioactivity per Mouse [MBq]	Injected Volume per Mouse [μL]	Radioactivity Concentration [MBq/mL]	Animals per Group
**Biokinetics**	24 h p.i.	0.25	30	8.3	5 (13 days after * TT)
	72 h p.i.				
	96 h p.i.	1	40	25	
	120 h p.i.				
Therapy	RIC 5 d after TT *	Control	50		5
		1		20	5
		2		40	6
		3		60	4
	RIC 2 d after * TT	Control			
		1		20	4
		2		40	
		3		60	

* TT: tumor transplantation.

## Data Availability

The original contributions presented in the study are included in the article/Supplementary Materials, further inquiries can be directed to the corresponding author.

## Supplementary Materials

The following supporting information can be downloaded at: https://www.mdpi.com/article/10.3390/ijms251910638/s1.
