# Supplementary material for: Localization and Tumor Growth Inhibition of I-131-Labeled Monoclonal Antibody ERIC1 in a Subcutaneous Xenograft Model of Small Cell Lung Cancer in SCID Mice
Source: Int J Mol Sci. 2024 Oct 2;25(19):10638. doi: 10.3390/ijms251910638 (PMC11477417; doi:10.3390/ijms251910638)
Supplement: Supplementary file 1 [file ijms-25-10638-s001.zip › ijms-3191509-supplementary.pdf]

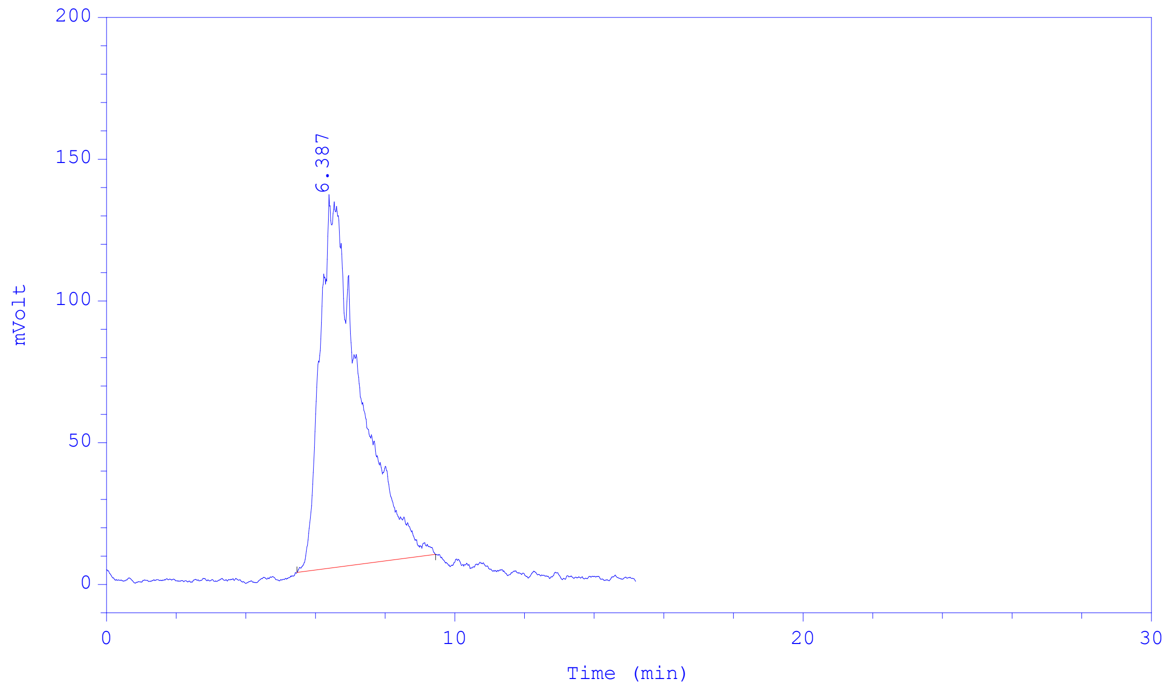

Representative HPLC radiochromatogram: The labeled antibody eluted between 6 and 7 minutes, while free [^{131}I]I-iodide typically elutes between 10 and 11 minutes. No free I-131 was detected.
